# Sedation-Induced Burst Suppression Predicts Positive Outcome Following Traumatic Brain Injury

**DOI:** 10.3389/fneur.2021.750667

**Published:** 2021-12-22

**Authors:** Joel Frohlich, Micah A. Johnson, David L. McArthur, Evan S. Lutkenhoff, John Dell'Italia, Courtney Real, Vikesh Shrestha, Norman M. Spivak, Jesús E. Ruiz Tejeda, Paul M. Vespa, Martin M. Monti

**Affiliations:** ^1^Department of Psychology, University of California, Los Angeles, Los Angeles, CA, United States; ^2^Department of Neurosurgery, Brain Injury Research Center (BIRC), UCLA Brain Injury Research Center, David Geffen School of Medicine, University of California, Los Angeles, Los Angeles, CA, United States

**Keywords:** traumatic brain injury (TBI), burst suppression, barbiturates, coma, disorders of consciousness, EEG biomarker, Glasgow Coma Scale (GCS), Glasgow Outcome Scale extended (GOSe)

## Abstract

While electroencephalogram (EEG) burst-suppression is often induced therapeutically using sedatives in the intensive care unit (ICU), there is hitherto no evidence with respect to its association to outcome in moderate-to-severe neurological patients. We examined the relationship between sedation-induced burst-suppression (SIBS) and outcome at hospital discharge and at 6-month follow up in patients surviving moderate-to-severe traumatic brain injury (TBI). For each of 32 patients recovering from coma after moderate-to-severe TBI, we measured the EEG burst suppression ratio (BSR) during periods of low responsiveness as assessed with the Glasgow Coma Scale (GCS). The maximum BSR was then used to predict the Glasgow Outcome Scale extended (GOSe) at discharge and at 6 months post-injury. A multi-model inference approach was used to assess the combination of predictors that best fit the outcome data. We found that BSR was positively associated with outcomes at 6 months (*P* = 0.022) but did not predict outcomes at discharge. A mediation analysis found no evidence that BSR mediates the effects of barbiturates or propofol on outcomes. Our results provide initial observational evidence that burst suppression may be neuroprotective in acute patients with TBI etiologies. SIBS may thus be useful in the ICU as a prognostic biomarker.

## Introduction

Biomarkers of recovery are greatly needed in coma following traumatic brain injury (TBI) for prognosis and to inform crucial medical and management decisions, including withdrawal of life-sustaining care (1). Associating electroencephalogram (EEG) patterns such as burst-suppression with patient outcomes may allow for the discovery of prognostic biomarkers. EEG burst-suppression patterns are commonly observed in coma patients, but are challenging to interpret since, in different contexts, their presence may be considered benign, therapeutic, or life-threatening (2). Pharmacologically-induced burst-suppression (i.e., deep anesthesia) is generally regarded as safe and is often induced intentionally in the intensive care unit (ICU) to reduce the cerebral metabolic rate and intracranial pressure (ICP) in patients with severe traumatic brain injury (TBI) (3, 4) and/or nonconvulsive status epilepticus (5). However, even in a therapeutic context, Niedermeyer and colleagues described burst-suppression as “incompatible with normal brain functioning” and therefore harmful, albeit allowing for vast recovery (2). Yet, to date, no study has specifically investigated the association between this EEG pattern and chronic outcome in patients surviving moderate-to-severe TBI. In what follows, we examine sedation-induced burst-suppression (SIBS) and assess the association between EEG burst-suppression ratio (BSR) at the acute time-point (i.e., ~3 weeks post-injury) and global outcome, as measured by the Glasgow Outcome Scale extended (GOSe) (6), at discharge from the hospital (2–51 days post-injury) and at a chronic (~6 month) follow-up.

## Methods

### Subjects

Our sample consisted of 32 patients with usable data admitted at the UCLA Ronald Reagan University Medical Center Neuroscience/Trauma ICU from December 2015 to January 2020 following moderate-to-severe TBI. Inclusion criteria were an admission Glasgow Coma Scale (GCS) (7) score ≤ 8 or an admission GCS score of 9–14 with computed tomography (CT) evidence of intracranial bleeding. Exclusion criteria were a GCS > 14 with non-significant head CT, history of neurologic disease or TBI, and brain death. Our sample size was not determined a priori but rather constrained by the number of available patients. For a flowchart illustrating how many patients were excluded at each step of the data processing pipeline and why, see Supplementary Figure 1. The study was approved by the UCLA Institutional Review Board; informed assent and consent were obtained per state regulations. Multiday EEG was recorded continuously (Cz reference) throughout each patient's stay in the ICU. Data were acquired using Nicolet (Natus Medical, Inc., Pleasanton, CA, USA) or Moberg ICU Solutions (Moberg Research, Inc., Ambler, PA, USA) EEG systems. Persyst software (Persyst Development Corporation, Solana Beach, CA, USA) was used to de-identify and export EEG data in European Data Format (EDF) to MATLAB (version R2019b, The MathWorks, Inc., Natick, MA, USA) for analysis. Anesthetic medications, administered to manage symptoms and/or reduce cerebral metabolism, were noted on a daily basis and sorted into two categories (barbiturates and propofol). In particular, burst suppression was induced using barbiturates in patients with therapy refractory ICP elevation, following evidence (4) showing efficacy for treatment of ICP, and to treat increased brain edema.

### Behavioral Assessments

During ICU hospitalization, behavioral assessments were performed several times daily using the GCS. Global outcomes were assessed with the GOSe in-person interview protocol at hospital discharge 2–51 days post-injury (22 ± 10 days, mean ± std) and either in-person or by phone to patients and/or family members at a chronic follow-up 161 – 318 days post-injury (193 ± 34.5 days, mean ± std; 4 patients had chronic assessments more than 7 months post-injury, see Table 1).

**Table 1:** Patients demographics, assessment scores, and medications.

**Patient**	**Age bin (at injury)**	**sex**	**EEG system**	**Max BSR**	**Max GCS score**	**Discharge GOSe**	**Days to discharge GOSe**	**Chronic GOSe**	**Days to chronic GOSe**	**Barbiturates**	**Propofol**	**Usable EEG sections**	**Total EEG sections**	**Percent of sections used**
1	18 – 25	M	Nicolet	0.9997	8	3	17	7	183	TRUE	FALSE	6	6	100
2	25 – 40	F	Nicolet	0.9996	14	3	2	7	188	TRUE	TRUE	4	5	80
3	25 – 40	M	Nicolet	0.9996	8	3	24	8	180	TRUE	FALSE	7	7	100
4	55 – 70	F	Nicolet	0.9323	14	3	23	Missing	N/A	TRUE	FALSE	6	6	100
5	25 – 40	M	Nicolet	0.9313	4	3	38	3	246	TRUE	TRUE	6	9	66.7
6	40 – 55	M	Nicolet	0.9275	7	3	29	5	203	TRUE	FALSE	7	7	100
7	18 – 25	M	Moberg	0.8747	11	3	30	6	179	TRUE	FALSE	5	6	83.3
8	18 – 25	M	Nicolet	0.8708	10	3	24	7	181	TRUE	FALSE	4	6	66.7
9	25 – 40	M	Nicolet	0.4674	4	2	23	3	183	TRUE	FALSE	6	7	85.7
10	55 – 70	M	Nicolet	0.422	8	2	16	Missing	N/A	FALSE	TRUE	5	6	83.3
11	40 – 55	M	Nicolet	0.148	11	3	35	2	172	FALSE	TRUE	6	6	100
12	40 – 55	M	Nicolet	0.1467	14	3	33	5	189	FALSE	TRUE	5	5	100
13	25 – 40	M	Nicolet	0.1376	10	2	23	3	179	FALSE	TRUE	6	6	100
14	55 – 70	F	Nicolet	0.0555	10	1	30	1	N/A	FALSE	FALSE	6	7	85.7
15	70 – 85	M	Nicolet	0.0548	15	3	13	6	182	FALSE	FALSE	3	3	100
16	40 – 55	M	Nicolet	0.0316	11	2	24	3	182	FALSE	FALSE	3	4	75
17	40 – 55	M	Moberg	0.0254	14	4	20	5	184	TRUE	TRUE	2	3	66.7
18	25 – 40	M	Nicolet	0.0193	14	3	4	7	179	FALSE	FALSE	3	3	100
19	18 – 25	M	Nicolet	0.0181	14	3	5	8	184	FALSE	FALSE	1	1	100
20	25 – 40	M	Nicolet	0.017	7	2	24	2	161	FALSE	TRUE	8	8	100
21	70 – 85	M	Nicolet	0.014	14	3	8	5	177	FALSE	TRUE	3	5	60
22	55 – 70	M	Nicolet	0.0098	14	3	24	3	224	FALSE	TRUE	7	7	100
23	25 – 40	M	Nicolet	0.0095	6	1	17	1	N/A	FALSE	TRUE	5	5	100
24	25 – 40	M	Nicolet	0.0076	9	2	20	2	177	FALSE	TRUE	4	4	100
25	40 – 55	M	Nicolet	0.0055	14	4	14	6	174	FALSE	FALSE	3	3	100
26	25 – 40	M	Nicolet	0.0039	10	3	20	6	182	FALSE	FALSE	5	6	83.3
27	25 – 40	M	Nicolet	0.0028	10	2	26	3	177	FALSE	TRUE	2	6	33.3
28	55 – 70	F	Nicolet	0.002	14	3	11	3	181	FALSE	TRUE	3	5	60
29	55 – 70	M	Nicolet	0.0015	10	2	23	3	279	FALSE	TRUE	7	8	87.5
30	55 – 70	M	Nicolet	0.0008	14	3	18	7	318	FALSE	TRUE	3	3	100
31	18 – 25	M	Nicolet	0.0001	6	2	24	3	164	FALSE	FALSE	4	4	100
32	18 – 25	F	Nicolet	0	8	2	51	3	192	FALSE	TRUE	2	2	100

*Our cohort consisted of 32 patients (5 female), age at injury = 41 ± 17 years (mean ± STD). Age at injury is reported above in bins to protect patients' anonymity. Patients in our sample were predominantly male (84%), reflecting a frequently reported greater risk in males for TBI (8, 9). Medications were coded as dichotomous variables, according to whether the patient was administered a drug of this category (barbiturates or propofol) on the day of maximum BSR (TRUE = the patient received the drug on the day of maximum BSR; FALSE = the patient did not receive the drug on the day of maximum BSR). Time to chronic follow up is reported in number of days post-injury; note that patients who scored a 1 on the chronic GOSe were deceased and therefore the time to chronic follow up is not applicable*.

### EEG Section Selection

**Figure 1:**
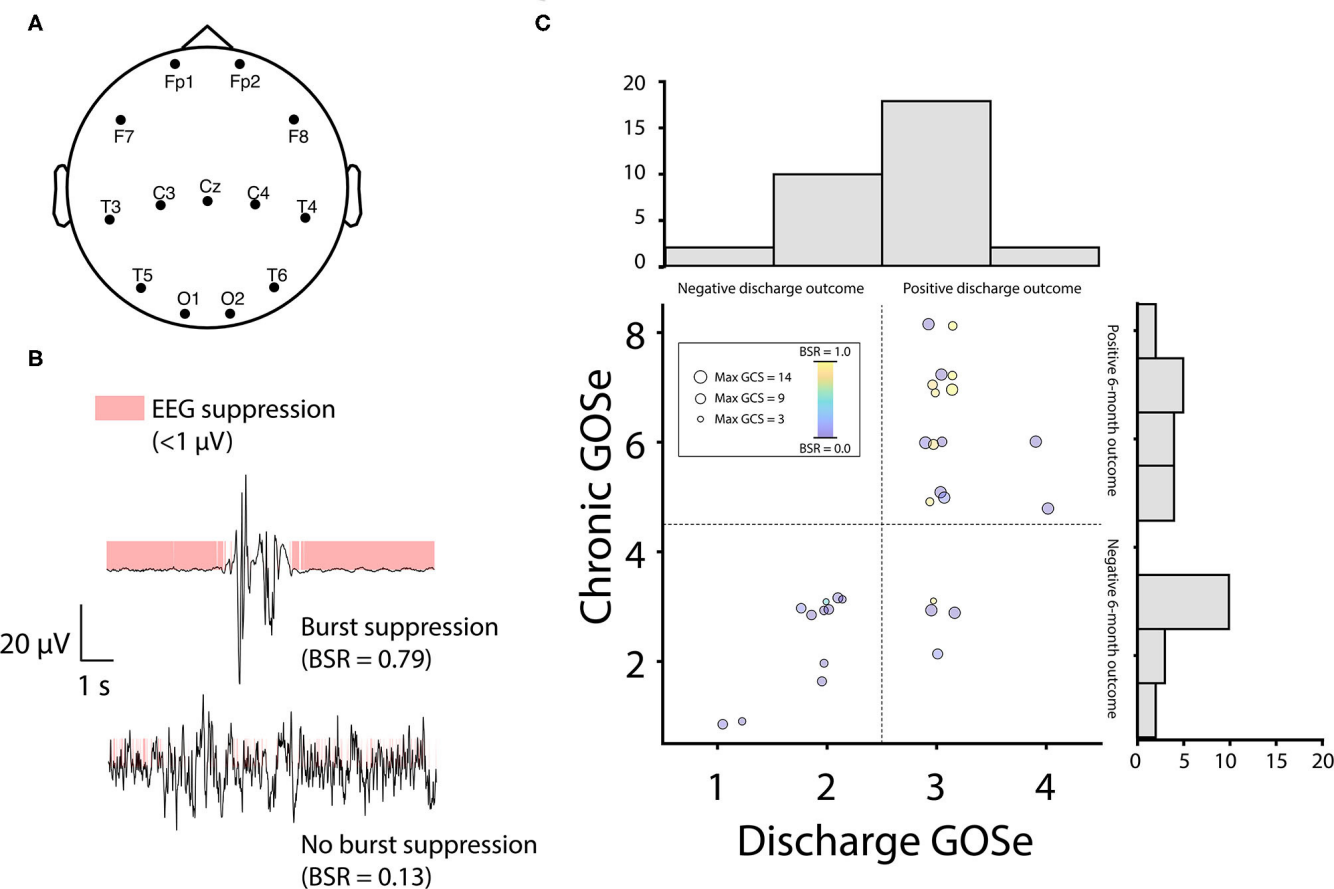
EEG burst-suppression positively predicts outcomes at chronic follow up. **(A)** EEG signals were recorded from 13 channels common to all patients. Channel placement was frequently modified from standard positions (shown above) to accommodate bone flaps and injury sites in individual patients. **(B)** The EEG burst suppression ratio (BSR) was computed as the proportion of clean signal with a rectified amplitude < 1 μV (mode across channels). **(C)** Outcomes were assessed using the Glasgow Outcome Scale extended (GOSe) and were divided at discharge and chronic timepoints using median splits (dotted lines, GOSe scores are scattered with jitter to avoid overlap). Patients with burst-suppression (red circles) were overrepresented in the upper half of each median split (blue circles = no burst-suppression; circle size is proportional to patient's highest Glasgow Coma Scale score). Note that 2 patients missing chronic GOSe scores (Patients 4 and 10, Table 1) are not shown in the scatter plot but are represented in the discharge GOSe histogram. GOSe scores at discharge showed low spread, with most patients either scoring 2 (vegetative state) or 3 (low severe disability). Similarly, chronic GOSe scores showed a bimodal distribution with peaks flanking a large dip at 4 (upper severe disability). Owing to this fact, we opted for a median split on scores (discharge: 1-2 vs. 3-4; chronic: 1-4 vs. 5-8) to create dichotomous outcome variables for logistic regression. In the upper half of both the discharge and chronic GOSe median split, 40% of patients displayed BSR > 0.5, vs. 0% (discharge) and 6.7% (chronic) of patients in the lower half of the GOSe distribution.

Our analysis of burst suppression was data-driven (i.e., we measured burst suppression using the BSR computed directly from EEG data rather than inferring bust suppression from other records). The goal of our EEG selection was therefore to include the most discontinuous EEG from all patients by extracting those sections of EEG that corresponded to low behavioral responsiveness. If burst suppression was not used in patients or they otherwise did not show discontinuous EEG, their data were still included and benefited the analysis by increasing the between-subjects variance in BSR. To analyze patients during periods of minimal responsiveness when SIBS was used, we extracted 10 min of EEG from 13 channels common to all patients (Figure 1A) from timepoints corresponding to low GCS scores, with EEG sections spaced a minimum of 24 h apart (Supplementary Figure 2). Note that although low GCS scores do not necessarily imply sedation, we expected sedation to only coincide with low GCS scores and therefore used GCS scores to guide EEG extraction. Specifically, EEG sections were extracted by sorting each patient's GCS scores from low to high, appending the lowest score to a second list, and then crawling down the first list of GCS scores and adding each timepoint that was at least 24 h from any timepoint on the second list to the second list. EEG sections were then extracted from the second list's timepoints.

### EEG Data Processing

EEG extraction covered time points most likely to contain burst suppression (as determined by low GCS, see above) and was unbiased with respect to patient outcomes. Data were extracted from 13 channels common to all patients (Figure 1A), resulting in 215 EEG sections from 40 patients that entered preprocessing (corrupted data from one patient were rejected prior to preprocessing). After importing, all EEG data were re-referenced to average and bandpass filtered 0.5–45 Hz to attenuate low frequency drift and high frequency muscle artifacts and line noise. EEG data were further artifact reduced using a combination of manual exclusion of bad segments and independent component analysis (ICA) (10). EEG sections corresponding to excessive noise or disconnected equipment were discarded.

### Burst Suppression Ratio

We computed the BSR of usable EEG sections as the proportion of the total data with an amplitude < 1 μV [in line with the range recommended by Westover and colleagues (11); see Figure 1B and Supplementary Figure 3]. Data were binarized using this threshold (as chosen according to the method described below) after rectifying the signal (12), and the modal binary value across channels was used to construct a single time series on which the proportion was based (Figure 1B). Higher proportions correspond to more discontinuous EEG patterns and lower proportions correspond to more continuous EEG patterns. Note that we did not compute time spent in burst suppression, as we were only interested in the greatest depth of burst suppression (i.e., maximum BSR) for each patient and, moreover, computing total time spent in burst suppression would be impractical as it would require processing and analyzing several days of EEG recordings for each patient.

To determine the proper threshold for computing the BSR, we performed a sensitivity analysis by computing BSR on all usable EEG sections from all 32 patients in Table 1, plus 2 additional patients excluded from other analyses due to missing GOSe (74-year-old male) and medication (23-year-old female) data whose EEG data were nonetheless perfectly suitable for this particular analysis. This yielded 154 EEG sections from 34 patients. We used several different thresholds (1, 5, and 10 μV; Supplementary Figure 3) and then compared the resulting BSR values to 1) the concurrent GCS score and 2) the log-transformed kurtosis of voltage amplitudes, which should scale linearly with BSR (with the exception of BSR = 1.0 or isoelectric EEG, in which the complete absence of EEG bursts yields low kurtosis values).

### Statistical Analysis

In order to relate each patient's greatest depth of burst suppression to global outcome, we extracted the maximum BSR across all EEG sections for each patient. Owing to their non-normal and non-linear characteristics (13), GOSe scores were binarized according to a median split (Figure 1C). Model selection and averaging were performed using the Multi-Model Inference package in R (version 3.6.2) (14). Multiple logistic regression models were fitted to predict outcome using any of the following: maximum BSR, maximum GCS score, sex, and age at injury (all models included an intercept). We covaried for the maximum GCS given that it reflects the patient's overall behavioral recovery in the ICU. Note that the maximum GCS was computed from the patient's entire ICU stay and not just those timepoints for which EEG data were extracted (which corresponded to GCS minima rather than maxima). Model averaged parameters were derived using all models within 5 small-sample corrected Akaike information criterion (AICc) units of the leading model for each dependent variable (discharge and chronic outcome, see Table 2).

**Table 2:** Multiple logistic regression models.

**Outcome**	**Intercept**	**Age at injury**	**Maximum BSR**	**Sex**	**Maximum GCS**	**df**	**logLik**	**AICc**	**Delta**	**Weight**
**Discharge**	**−28.610**	**−0.204**	**25.348**	**NA**	**3.332**	**4.000**	**−3.770**	**17.021**	**0.000**	**0.452**
**Discharge**	**−18.230**	**NA**	**13.866**	**NA**	**1.572**	**3.000**	**−5.366**	**17.588**	**0.567**	**0.340**
**Discharge**	**−28.600**	**−0.204**	**25.339**	**−0.154**	**3.330**	**5.000**	**−3.770**	**19.847**	**2.826**	**0.110**
**Discharge**	**−18.123**	**NA**	**13.693**	**−1.171**	**1.574**	**4.000**	**−5.293**	**20.068**	**3.047**	**0.098**
Discharge	−3.911	NA	NA	NA	0.437	2.000	−15.907	36.227	19.206	0.000
Discharge	−3.638	−0.038	NA	NA	0.561	3.000	−15.273	37.404	20.383	0.000
Discharge	−4.090	NA	NA	−1.111	0.473	3.000	−15.508	37.872	20.851	0.000
Discharge	−3.750	−0.038	NA	−1.045	0.590	4.000	−14.919	39.319	22.298	0.000
Discharge	−1.959	0.042	3.237	NA	NA	3.000	−17.222	41.301	24.280	0.000
Discharge	−0.057	NA	2.633	NA	NA	2.000	−18.588	41.589	24.568	0.000
Discharge	−2.008	0.046	3.301	−0.778	NA	4.000	−17.033	43.548	26.527	0.000
Discharge	−0.001	NA	2.673	−0.441	NA	3.000	−18.513	43.882	26.861	0.000
Discharge	0.511	NA	NA	NA	NA	1.000	−21.170	44.473	27.452	0.000
Discharge	−0.285	0.020	NA	NA	NA	2.000	−20.800	46.014	28.993	0.000
Discharge	0.531	NA	NA	−0.125	NA	2.000	−21.162	46.738	29.717	0.000
Discharge	−0.280	0.020	NA	−0.252	NA	3.000	−20.770	48.397	31.376	0.000
**Chronic**	**−13.414**	**NA**	**8.083**	**−5.818**	**1.084**	**4.000**	**−7.246**	**24.093**	**0.000**	**0.422**
**Chronic**	**−12.631**	**NA**	**7.917**	**NA**	**0.985**	**3.000**	**−8.980**	**24.883**	**0.790**	**0.284**
**Chronic**	**−13.169**	**−0.047**	**8.444**	**−6.289**	**1.253**	**5.000**	**−6.856**	**26.212**	**2.119**	**0.146**
**Chronic**	**−12.348**	**−0.054**	**8.340**	**NA**	**1.181**	**4.000**	**−8.311**	**26.222**	**2.130**	**0.146**
Chronic	−3.838	−0.059	NA	NA	0.590	3.000	−15.336	37.595	13.503	0.000
Chronic	−4.066	−0.065	NA	−2.285	0.663	4.000	−14.040	37.680	13.587	0.000
Chronic	−3.799	NA	NA	NA	0.359	2.000	−16.967	38.378	14.286	0.000
Chronic	−4.052	NA	NA	−2.029	0.409	3.000	−15.733	38.389	14.296	0.000
Chronic	−0.492	NA	2.075	NA	NA	2.000	−18.785	42.014	17.922	0.000
Chronic	−0.340	NA	2.199	−1.525	NA	3.000	−18.070	43.062	18.970	0.000
Chronic	−1.795	0.029	2.614	NA	NA	3.000	−18.155	43.233	19.140	0.000
Chronic	0.000	NA	NA	NA	NA	1.000	−20.794	43.732	19.639	0.000
Chronic	−1.921	0.035	2.866	−1.808	NA	4.000	−17.233	44.066	19.973	0.000
Chronic	0.154	NA	NA	−1.253	NA	2.000	−20.194	44.833	20.740	0.000
Chronic	−0.185	0.005	NA	NA	NA	2.000	−20.773	45.990	21.897	0.000
Chronic	−0.123	0.007	NA	−1.286	NA	3.000	−20.147	47.216	23.124	0.000

*For each outcome variable (discharge and chronic), 16 models were generated using combinations of predictors: age at injury, maximum BSR, sex, and maximum GCS (beta coefficients are reported above). We then averaged across all models within 5 small-sample corrected Akaike information criterion (AICc) units of the leading model (i.e., delta < 5) to derive model parameters. Bolded rows indicate best-fit models that were averaged to produce parameter estimates*.

### Mediation Analysis

Given that burst suppression was induced by sedatives, we asked whether BSR mediates effects of barbiturates and/or propofol on outcomes. Mediation analyses were performed in R (version 3.6.2) using the Mediation package (15). Specifically, we ran nonparametric mediation analyses using 1000 Monte Carlo simulation per analysis. Four separate analyses were run with barbiturates/propofol as the independent variable and discharge/chronic outcome as the dependent variable. In addition to these, we also ran analyses with BSR as the independent variable and medication as the mediator. Medications were coded as binary variables according to whether the patient was administered the medication on the same day as the patient's maximum BSR. Each analysis included a regression model predicting the mediator from the independent variable and another regression model predicting outcome from the independent variable and mediator; logistic regression was used when predicting binary variables. Analyses also controlled for each patient's maximum GCS score, as this was the only significant covariate in any analysis (see section Results).

### Missing Data Analysis

To investigate whether *n* = 8 patients who were excluded due to excessive noise, technical artifacts, or missing medication data (see Supplementary Figure 1 for an illustration of where each patient was excluded in the workflow) were different in their outcomes than patients whose data were retained, we compared the proportion of patients with outcomes above and below the median split cutoff for the GOSe at discharge (0 if < 3, otherwise 1) and chronic (0 if < 5, otherwise 1) assessments using a chi-squared test.

## Results

Prior to analysis, data from 8 patients that had entered preprocessing were excluded entirely (Supplementary Figure 1) for the following reasons: 1) data quality (e.g., due to disconnected equipment or unremovable technical artifacts; 6 patients), 2) no GOSe assessments (1 patient), 3) no medication data (1 patient). Of the 32 remaining patients (Table 1), 2 patients were missing chronic GOSe scores and excluded from its prediction; these patients were also excluded from mediation analyses. Patients in our sample were predominantly male (84%), reflecting a frequently reported greater risk in males for TBI (8, 9). EEG processing yielded 4.6 ± 1.8 usable EEG sections per patient, or 88 ± 17% of extracted EEG sections (mean ± std).

### Burst Suppression Ratio Threshold

Of three different voltage threshold values (1, 5, and 10 μV), we selected a 1 μV threshold given that this threshold 1) only yielded high BSR values for EEG sections coinciding with GCS = 3 (i.e., the lowest possible responsiveness score) and 2) yielded BSR values that correlated strongly with the log_10_(kurtosis) of the signal (*r* = 0.88) (see Supplementary Figure 3 for further details).

### EEG Patterns

Ten patients (31%) displayed visual evidence of an isoelectric or burst suppression EEG (Supplementary Figure 4); however, BSR was treated as a continuous variable and was not thresholded or otherwise transformed to create categories of burst suppression and non-burst suppression. EEG patterns followed an expected progression from continuous EEG (low BSR) to burst suppression (high BSR), followed by isoelectric EEG (BSR ~ 1). See Supplementary Figure 4 for representative examples of EEG occurring at maximum BSR for all patients. The proportion of patients sedated with barbiturates increased with BSR (Supplementary Figure 5), reaching 90% at BSR > 0.15 and 100% at BSR > 0.45. EEG channels showed nearly perfect agreement regarding whether the rectified signal exceeded the 1 μV threshold as measured using intraclass correlations (ICCs) derived from each patient's EEG section corresponding to the maximum BSR (mean ICC = 0.999, min ICC = 0.992, max ICC = 1.0).

### Multi-Model Inference

GOSe scores were not correlated with number of post-injury days to assessment (discharge: *r* = −0.24, *P* = 0.18; chronic: *r* = 0.05, *P* = 0.79). Using multi-model inference, model parameters were averaged across four models each to predict discharge and chronic outcomes. No variable significantly predicted outcome at discharge. Maximum BSR (*P* = 0.022) and maximum GCS (*P* = 0.011) significantly predicted outcome at 6 months (Table 3). Next, medications were examined as binary variables indicating whether the patient was given the medication on the day of maximum BSR. No significant mediation effects were detected after modeling barbiturates and propofol separately as independent variables and BSR as a mediator variable and vice versa.

**Table 3:** Model averaged parameters.

**Outcome**	**Predictor**	**Beta estimate**	**Odds ratio**	**Standard error**	**Adjust standard error**	***Z*-value**	***p*-value**
Discharge	Intercept	−24.05	3.60E-11	15.38	16.00	1.50	0.13
Discharge	Max BSR	20.30	6.52E+08	13.74	14.25	1.42	0.15
Discharge	Max GCS	2.56	12.93	1.95	2.02	1.27	0.21
Discharge	Age at injury	−0.20	0.82	0.17	0.18	1.13	0.26
Discharge	Sex	−0.63	0.53	7.70	8.06	0.08	0.94
Chronic	**Intercept**	**−13.00**	**2.26E-06**	**4.84**	**5.08**	**2.56**	**0.01**
Chronic	**Max BSR**	**8.13**	**3.38E+03**	**3.38**	**3.55**	**2.29**	**0.02**
Chronic	**Max GCS**	**1.09**	**2.99**	**0.41**	**0.43**	**2.55**	**0.01**
Chronic	Age at injury	−0.05	0.95	0.05	0.06	0.89	0.38
Chronic	Sex	−5.94	2.63E-03	6.50	6.82	0.87	0.38

*Using a multi-model interference approach, we averaged across models with the best fit to produce estimates of the above parameters. Bolded rows above correspond to significant (p < 0.05) predictors. No predictors significantly predicted outcome at discharge. The maximum burst suppression ratio (BSR), maximum Glasgow Coma Scale (GCS) score, and model intercept significantly predicted outcome at chronic follow up*.

### Missing Data Analysis

We compared outcomes from excluded patients (*n* = 8, see Supplementary Figure 1) to all patients with discharge GOSe scores (*n* = 32) and chronic GOSe scores (*n* = 30). In the case of discharge outcomes, 62.5 and 75% of patients fell above the median split (i.e., GOSe ≥ 3) in the retained and excluded samples, respectively. In the case of chronic outcomes, 50% of patients fell above the median split (i.e., GOSe ≥ 5) in both the retained and excluded samples. Proportions of outcomes above the median split did not significantly differ between retained and excluded patients at discharge (χ^2^ = 0.44, *P* =0.51) or chronic (χ^2^ = 0, *P* = 1.0) assessments.

## Discussion

In this study, we show that the use of SIBS in the ICU is associated with favorable outcome (i.e., GOSe ≥ 5) at > 5 months post-injury in TBI patients, after controlling for patients' maximum behavioral recovery (i.e., highest GCS score) in the ICU. Moreover, while all patients with SIBS were administered barbiturates (see Supplementary Figure 5), we did not find evidence that BSR mediated an effect of sedatives on outcome. Our results suggest that the use of SIBS in the setting of moderate-to-severe TBI should not automatically imply a poor prognosis, and may in fact be a biomarker of the brain's ability to respond to decreased metabolic demand.

Our finding that SIBS predicts chronic outcomes is particularly important in light of our recent finding that other EEG variables, such as oscillatory power and the presence/absence of power spectral peaks, do not predict chronic outcomes in the same cohort of patients with moderate-to-severe TBI (16). Our current analysis—which examined a subset of this cohort—taken together with our other recent findings cited above, demonstrates that SIBS predicts chronic outcomes in TBI better than conventional models, such as the ABCD model of mesocircuit recovery (16), which infers thalamocortical integrity from spectral peaks and does not explicitly take EEG burst suppression into account (17, 18). Furthermore, our results herein were achieved using sparse spatial sampling (i.e., 13 channels) with clinical, rather than research-grade, EEG, and we observed near-perfect correlations in the presence/absence of suppression between EEG channels, with an average ICC of 0.999 across patients. The foregoing suggests that our results are not dependent on spatial location and could be reproduced using many other EEG systems, including montages as simple as a single bipolar channel.

One implication of our findings is that coma recovery may follow one of two main trajectories. The first trajectory is the previously reported association between early recovery, as captured by the GCS, and better outcome (19, 20). The second trajectory is characterized by deep burst-suppression coma and a state of low maximum responsiveness in the ICU. Because this trajectory is also associated with positive outcomes, one may infer that SIBS is likely, from a mechanistic standpoint, neuroprotective and, from an observational standpoint, a biomarker of later recovery that may inform decisions to sustain care, contrary to published guidelines in other coma etiologies (21–23). Along these lines, a randomized multi-site study conducted several decades ago by Eisenberg and colleagues (4) demonstrated that treatment by high-dose barbiturates acutely improves ICP in patients with severe head injury by a factor of at least 2:1, increasing to 4:1 after controlling for prerandomization cardiac factors.

### Study Limitations

Several possible limitations of our study should be noted. 1) It cannot be ruled out that our method of extracting local portions of EEG may have missed burst-suppression in some patients. However, our sampling was unbiased such that there is no reason to believe that our sensitivity varied with patients' outcomes. 2) After exporting EEG files to EDF format to be analyzed for our study, it was brought to our attention that discontinuities in EEG recordings (e.g., when EEG equipment is temporarily disconnected) may not be reflected in timestamps. Thus, it is possible EEG may have been sampled at times asynchronously to the targeted portions defined by GCS scores. However, we observed that burst suppression/muscle activity coincided with low/high GCS scores, suggesting that GCS and EEG data were well aligned. 3) Because we did not have access to the time of day at which medications were given, it was not possible to confirm that anesthetic drugs were administered before burst suppression in EEG. However, it is likely that burst suppression was sedative-induced, given the causal relationship between sedatives and burst suppression and the close temporal proximity of the two events.

## Conclusions

SIBS might be a candidate biomarker of prognosis that relates positively with desirable outcomes in coma recovery following TBI. Future work is needed to clarify the neural mechanisms underlying this association and to determine why this same EEG pattern is, in other etiologies, generally associated with the opposite (i.e., poor) outcome (2, 24). Furthermore, the extent to which the therapeutic effect of SIBS is dose-dependent remains unknown and will require a future prospective study.

## Data Availability Statement

The datasets presented in this article are not readily available because researchers seeking to access data must first complete a material transfer agreement with UCLA. Requests to access the datasets should be directed to Joel Frohlich, joelfrohlich@gmail.com.

## Ethics Statement

The studies involving human participants were reviewed and approved by University of California Los Angeles Institutional Review Board. Informed consent for participation was obtained from patients' family members.

## Author Contributions

JF and MM conceived of the idea for this study and wrote the original draft of the manuscript. CR, VS, and JR collected and curated the data. EL and NS organized behavioral and medication data from patients. JD provided computing resources. JF processed and analyzed the data and also generated figures and tables. MJ and DM contributed to the statistical analysis. PV and MM acquired funding and supervised the project. All authors contributed to reviewing and editing the manuscript.

## Funding

This work was supported by grants from the Tiny Blue Dot Foundation (MM), the National Institute of Neurological Disorders and Stroke (PV, Grant Numbers NS058489, NS100064, and NS049471), and the State of California Neurotrauma Initiative (PV). Sponsors had no influence over any aspect of the work.

## Conflict of Interest

PV received personal fees from Ceribell and UCB Pharma outside the submitted work. The remaining authors declare that the research was conducted in the absence of any commercial or financial relationships that could be construed as a potential conflict of interest.

## Publisher's Note

All claims expressed in this article are solely those of the authors and do not necessarily represent those of their affiliated organizations, or those of the publisher, the editors and the reviewers. Any product that may be evaluated in this article, or claim that may be made by its manufacturer, is not guaranteed or endorsed by the publisher.
