# Supplementary material for: Sedation-Induced Burst Suppression Predicts Positive Outcome Following Traumatic Brain Injury
Source: Front Neurol. 2021 Dec 22;12:750667. doi: 10.3389/fneur.2021.750667 (PMC8727767; doi:10.3389/fneur.2021.750667)
Supplement: Supplementary file 1 [file Data_Sheet_1.docx]

**Supplementary Material**

**Supplementary Figures**

**
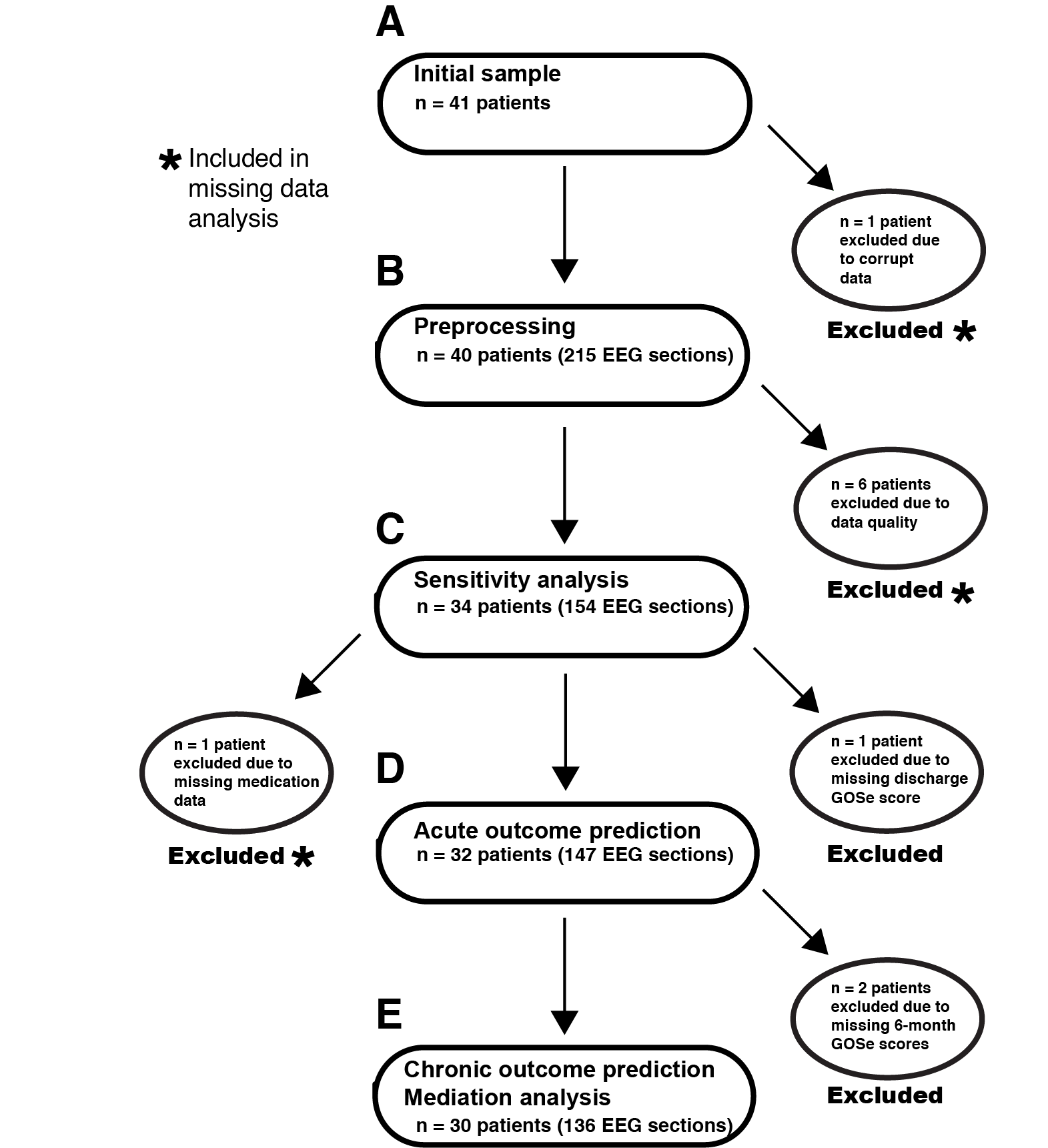
**

**Supplementary Figure 1: Flowchart of data pipeline.** Note that Glasgow Outcome Scale extended (GOSe) scores from patients with excluded data marked by an asterisk (*) were compared with GOSe scores from patients included in D (acute outcome) and E (chronic outcome) to determine if exclusion of these patients biased our sample. **(A)**. Prior to preprocessing, we excluded n = 1 patient with corrupt data. **(B)** Following preprocessing, we excluded n = 6 patients due to poor data quality (e.g., persistent artifacts, disconnected recording equipment, etc.). **(C)** After performing a sensitivity analysis and before proceeding to prediction of patient outcomes, we next excluded n = 1 patient missing medication data and n = 1 patient missing discharge GOSe data. **(D)** After prediction of discharge outcomes and before proceeding to prediction of chronic outcomes, we excluded n = 2 patients missing 6-month GOSe scores. **(E)** The remaining sample of n = 30 patients was used to predict chronic outcomes and entered into a mediation analysis.

**
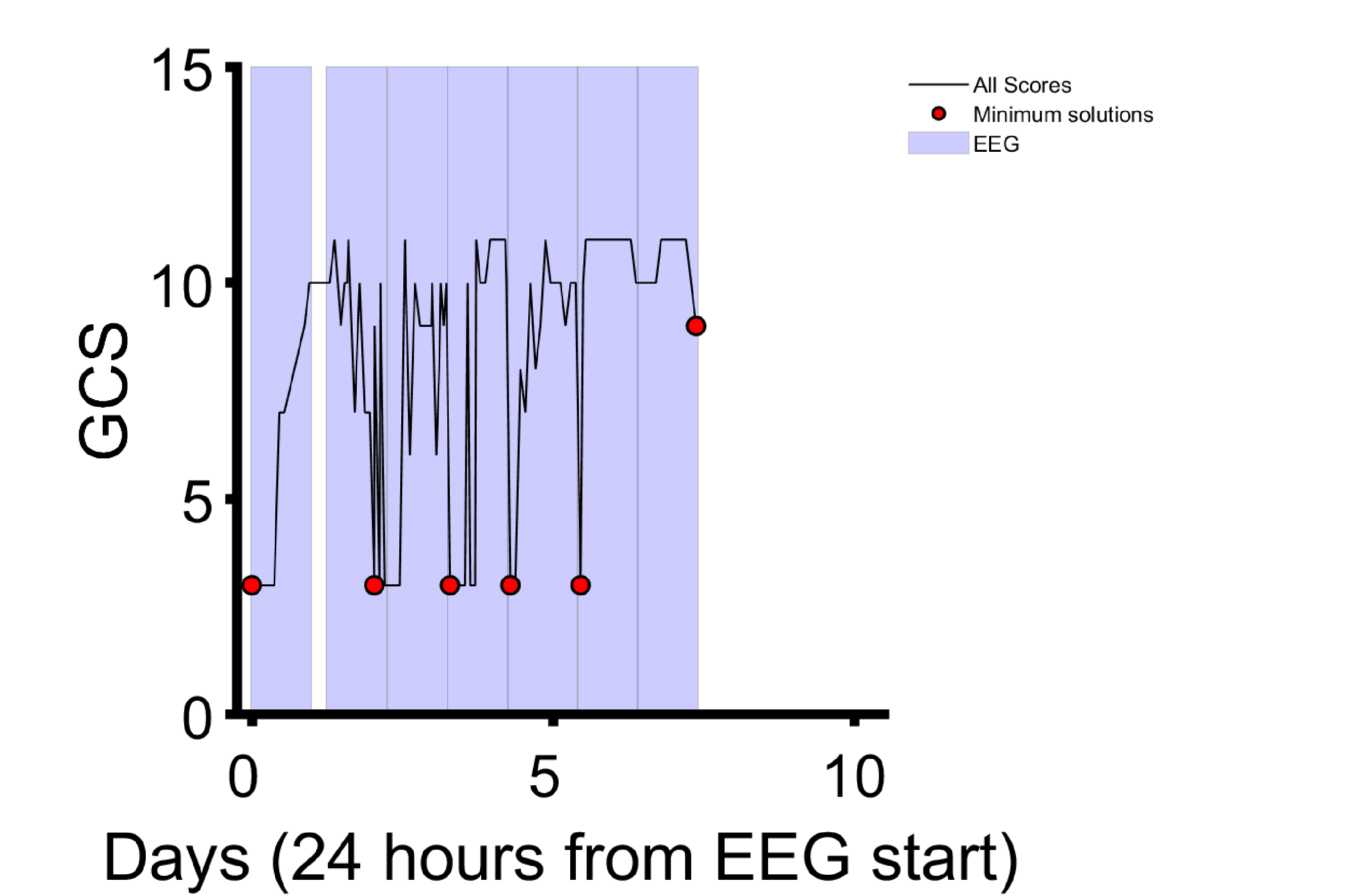
**

**Supplementary Figure 2: Example of EEG section selection.** 10-minute sections of multiday EEG recordings (purple highlights) were selected for analysis by extracting sections corresponding to each patient’s lowest level of responsiveness (as measured with the Glasgow Coma Scale or GCS) with a 24-hour buffer between extracted sections. GCS scores from patients, taken several times per day (black trace), were sorted low to high. Starting with the lowest and earliest GCS score, EEG sections (red circles) were extracted so long as they were spaced at least 24 hours from all other extracted EEG sections.


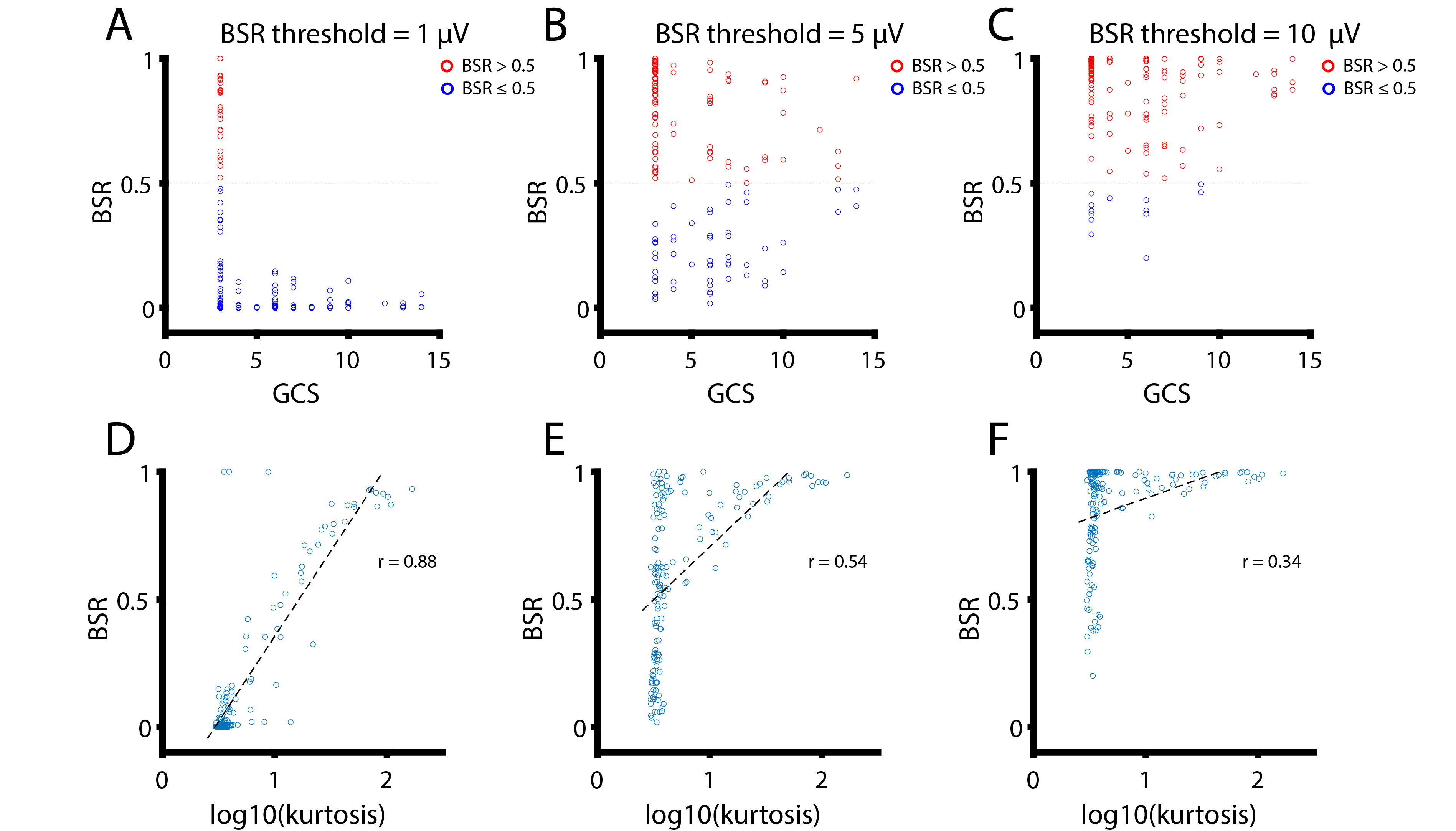


**Supplementary Figure 3: Sensitivity analysis for burst suppression ratio (BSR) threshold.** Three threshold values were tested to compute the BSR on 154 EEG sections: 1 μV. (first column), 5 μV (second column), and 10 μV (third column). To determine the best possible threshold, BSR values were compared to patient responsiveness using the Glasgow Coma Scale (GCS, top row) and the log-scaled kurtosis of EEG amplitudes (bottom row; this quantity is increased by burst events that create a leptokurtic distribution). Because burst suppression corresponds to a very deep state of coma/anesthesia, it should only be observable when patients are minimally responsive. Based on the above results, a threshold of 1 μV was chosen. **(A)** Using a 1 μV threshold, only EEG sections corresponding to a GCS of 3 (lowest possible score) displayed strong evidence of burst suppression (BSR > 0.5). **(B)** Using a 5 μV threshold, BSR > 0.5 is no longer specific to periods of low responsiveness but is instead observed for GCS scores as high as 14. **(C)** Using a 10 μV threshold, all specificity is lost; the vast majority of EEG sections show BSR > 0.5. **(D)** Using a 1 μV threshold, the log_10_ transformed kurtosis of EEG amplitudes shows a strong linear relationship with BSR (r = 0.88). Note three EEG sections with BSR ~ 1.0 at the top left corner that do not follow this relationship, as isoelectric EEG without bursts shows low kurtosis. **(E)** Using a 5 μV threshold, the linear relationship between log_10_ transformed kurtosis and BSR is considerably weaker (r = 0.54) compared with a 1 μV threshold. Many EEG sections display high BSR but low log_10_(kurtosis), suggesting many false positives. **(F)** Using a 10 μV threshold the linear relationship between log_10_ transformed kurtosis and BSR is nearly lost all together (r = 0.34) due to false positives.


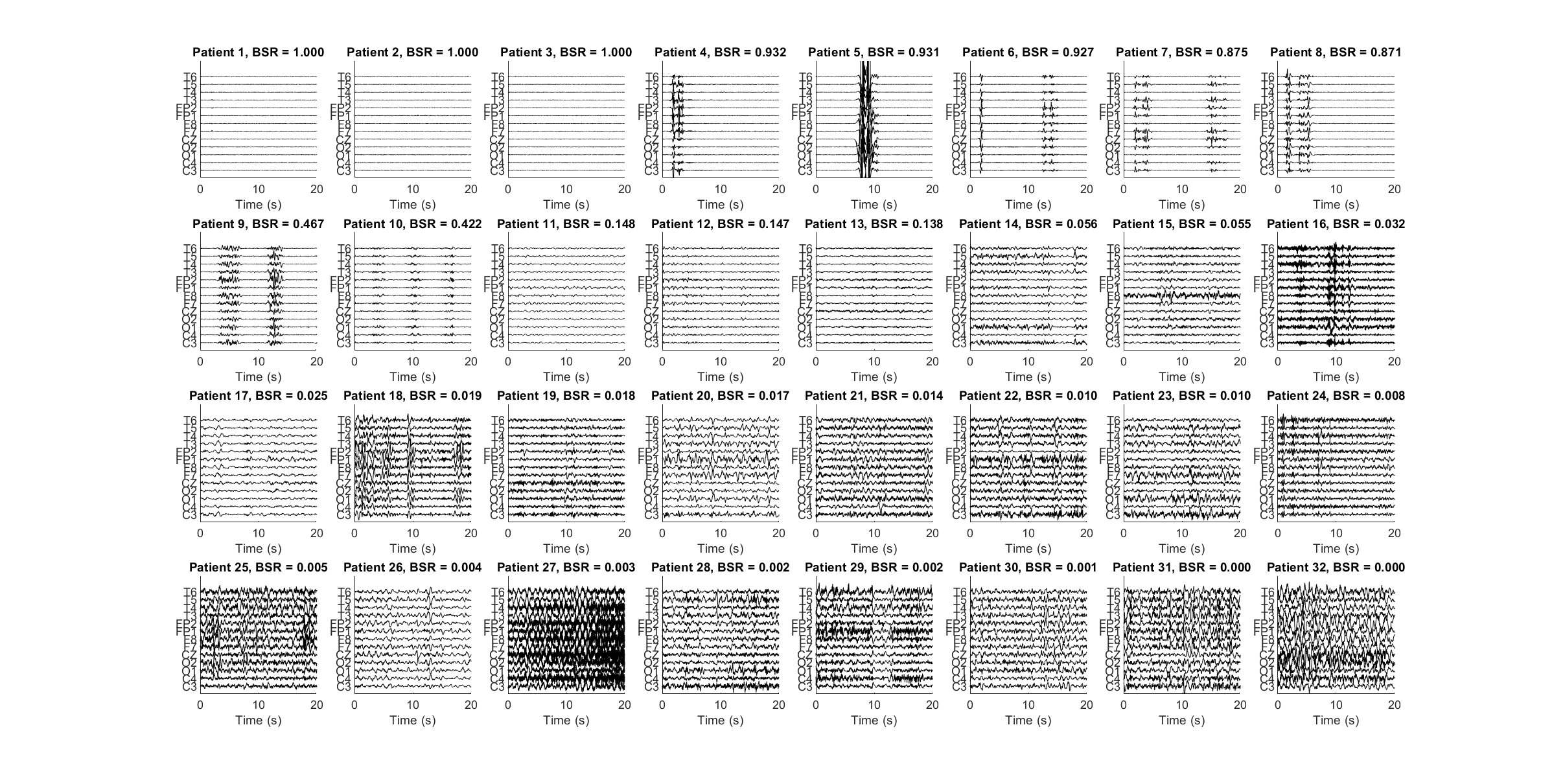


**Supplementary Figure 4: Representative EEG patterns occurring at time of maximum BSR for all patients.** EEGs are labeled according to the patient numbers in Table 1 of the main manuscript. As the burst suppression ratio (BSR) decreases, EEG evolves from isoelectric (patients 1 - 3) to burst suppression (patients 4 – 10) before finally showing continuous patterns (patients 11 – 32). All patients with BSR > 0.5 were sedated with barbiturates (see Fig. S5). Patient 9 (BSR = 0.467) was also sedated with barbiturates, and Patient 10 (BSR = 0.422) was sedated with propofol. Our analysis did not create categorical distinctions between burst suppression and non-burst suppression, but instead treated BSR as a continuous variable.


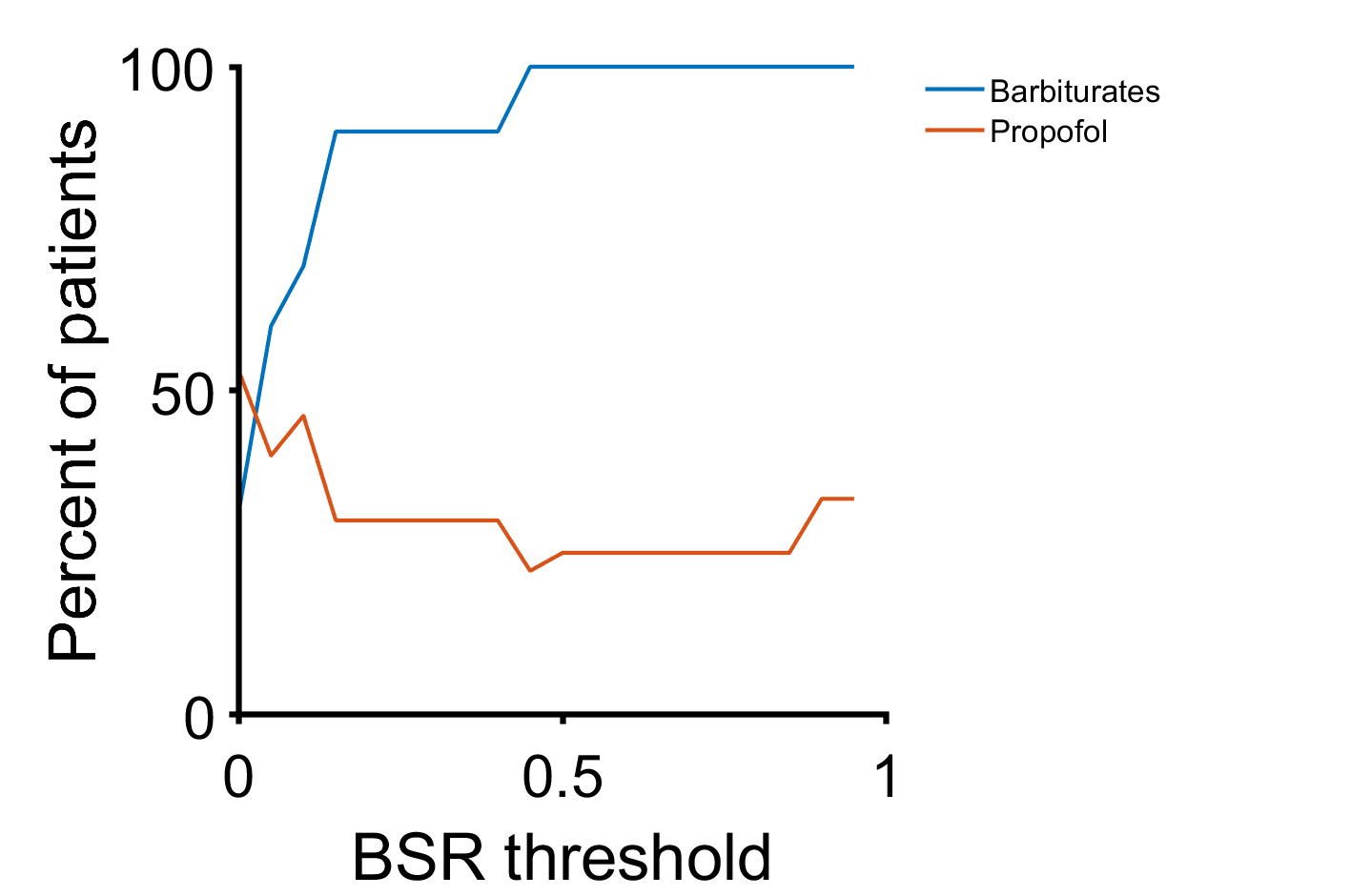


**Supplementary Figure 5: Percent of patients sedated with barbiturates or propofol as a function of bursts suppression ratio (BSR) threshold.** A threshold was applied to patients such that the proportion given barbiturates or propofol was computed for all patients with BSR > threshold. The proportion of patients sedated with barbiturates increases with the BSR threshold. All patients with BSR > 0.45 were given barbiturates on the day of maximum BSR. Burst suppression appeared to be largely induced by barbiturates rather than propofol. While slightly more than half (53%) of all patients were sedated with propofol on the day of maximum BSR, this proportion was smaller for those with higher BSRs.
